# Application of Machine Learning Models for the Early Detection of Metritis in Dairy Cows Based on Physiological, Behavioural and Milk Quality Indicators

**DOI:** 10.3390/ani15111674

**Published:** 2025-06-05

**Authors:** Karina Džermeikaitė, Justina Krištolaitytė, Ramūnas Antanaitis

**Affiliations:** Animal Clinic, Veterinary Academy, Lithuania University of Health Sciences, Tilžės Str. 18, LT-47181 Kaunas, Lithuania; justina.kristolaityte@lsmu.lt (J.K.); ramunas.antanaitis@lsmu.lt (R.A.)

**Keywords:** dairy cow, metritis, early diagnosis, machine learning, precision livestock farming

## Abstract

Metritis is one of the most common postpartum diseases in dairy cows, associated with impaired reproductive performance and substantial economic losses. In this study, we investigated the potential of machine learning (ML) techniques applied to physiological, behavioural, and milk quality parameters for the early detection of metritis in dairy cows during the postpartum period. A total of 2707 daily observations were collected from 94 cows in early lactation, of which 11 cows (275 records) were diagnosed with metritis. The dataset included daily measurements of body weight, rumination time, milk yield, milk composition (fat, protein, lactose), somatic cell count (SCC), and feed intake. Five classification models—partial least squares discriminant analysis (PLS-DA), random forest (RF), support vector machine (SVM), neural network (NN), and an Ensemble model—were developed using standardised features and stratified 80/20 training/test splits. To address class imbalance, model loss functions were adjusted using class weights. Models were evaluated based on accuracy, sensitivity, specificity, positive and negative predictive values (PPV, NPV), area under the receiver operating characteristic (ROC) area under the curve (AUC), and Matthews correlation coefficient (MCC). The NN model demonstrated the highest overall performance (accuracy = 96.1%, AUC = 96.3%, MCC = 0.79), indicating strong capability in distinguishing both healthy and diseased animals. The SVM achieved the highest sensitivity (90.9%), while RF and Ensemble models showed high specificity (>98%) and PPV. This study provides novel evidence that ML methods can effectively detect metritis using routinely collected, non-invasive on-farm data. Our findings support the integration of neural and Ensemble learning models into automated health monitoring systems to enable earlier disease detection and improved animal welfare. Although external validation was not performed, internal cross-validation demonstrated consistent performance across models, suggesting suitability for application in multi-farm settings. To the best of our knowledge, this is among the first studies to apply ML for early metritis detection based exclusively only automated herd data.

## Simple Summary

Metritis is a common uterine disease in dairy cows after calving and is associated with poorer reproductive performance, increased treatment costs, and decreased milk yield. Early diagnosis is critical but remains challenging in large herds due to limitations in manual observation. In this study, we applied machine learning models to analyse routinely collected on-farm data, including milk yield, milk composition, rumination time, and body weight, in order to identify early indicators of metritis. Five models were evaluated, with neural networks and support vector machines demonstrating the highest classification accuracy. Our findings indicate that it is possible to detect cows at risk using non-invasive parameters before the onset of severe clinical symptoms. This approach may support more timely and targeted treatment decisions by producers and veterinarians, thereby improving animal welfare and reducing unnecessary antibiotic usage.

## 1. Introduction

Metritis is one of the most common postpartum disorders in dairy cattle, negatively affecting animal welfare, reproduction, and farm profitability [1]. As an acute inflammatory condition with systemic consequences, metritis affects approximately 20–40% of postpartum dairy cows within the first 21 days in milk (DIM) [2]. This leads to reduced milk production, lower fertility, more early pregnancy losses, and higher risks of mortality and culling. Overall, metritis compromises animal welfare [3]. Early and reliable detection of metritis remains a considerable challenge, particularly in grazing or partially housed systems where frequent veterinary assessment is limited. Conventional diagnostic approaches are often subjective or applied late in the disease course [4,5,6].

Machine learning (ML) is a promising approach for improving disease detection using large datasets [7]. ML, a subfield of artificial intelligence, uses statistical methods to detect or forecast animal performance or disease events by analysing complex, multidimensional datasets [8]. ML algorithms support new ways of analysing dairy farm data collected through herd management systems and milk testing programmes [9]. ML methods are increasingly being used to enhance decision support tools across multiple areas of dairy science, including animal behaviour, nutrition, management, physiology, and reproduction. These techniques help identify important patterns and outcomes related to farm productivity. ML often performs better than traditional methods because it can detect complex, non-linear patterns without relying on assumptions like linearity [10]. The goal of ML is to replicate human-like extraction from large datasets. Accordingly, ML algorithms can identify clusters of relevant features, predict event onset, and continuously adapt based on the data they process [11].

Previous research has demonstrated the feasibility of integrating image, audio, and motion sensor data with algorithms to support decision making in dairy farming [6,12,13]. Recent advances in precision livestock farming have enabled the continuous monitoring of animal health using physiological, behavioural, and milk production parameters. Biosensor technologies contribute to improved cow health, economic efficiency, and overall herd productivity. Artificial intelligence (AI), particularly when combined with biosensor data, has shown promise in enhancing disease diagnosis, especially in periparturient disorders in dairy cattle. AI algorithms can analyse complex biosensor datasets to detect disease-associated patterns [14]. The emergence of new technologies for automatic behaviour recording facilitates the use of objective metrics, supporting the early detection and assessment of health problems at the individual animal level, with the potential to prevent or mitigate clinical disease [15]. When coupled with ML techniques, such data streams offer promising opportunities to develop early diagnostic tools [16]. ML algorithms can be used to build predictive models that identify cows at increased risk of developing clinical illness. By analysing behaviour frequently, ML can detect diseases faster than traditional methods, allowing for earlier treatment and reduced animal stress [15]. A predictive model capable of detecting cows at elevated risk of metritis, using routinely recorded on-farm data, may represent a valuable and practical tool for the dairy industry [17]. Morteza et al. [18] investigated various metabotypes of dairy cows throughout the transition period utilising decision tree, random forest, and naïve Bayes methodologies [18]. Moreover, Warner et al. [19] employed the K nearest neighbour, decision tree, and multilayer perceptron algorithms to differentiate aberrant behaviour in dairy cows suffering from subacute ruminal acidosis [19]. Recent studies have demonstrated the potential of deep learning in precision livestock health monitoring. For example, Wang et al. [20] applied Siamese networks to before-and-after images of feed troughs to estimate dairy cow feed intake, demonstrating how deep learning architectures can support non-invasive health-related monitoring [20]. Van Leerdam et al. [21] developed a deep learning model using behavioural sensor data to predict hypocalcaemia in dairy cows, achieving an area under the curve (AUC) of 0.71, and highlighting AI’s potential for early metabolic disorders [21]. Lee et al. [22] employed 3D point cloud data and deep learning to automatically measure cow body metrics, such as stature and teat length, providing a non-contact solution for continuous monitoring [22]. Additionally, Jia et al. [23] utilised key point tracking and deep learning to detect lameness from video footage, achieving 90.21% accuracy and highlighting the utility of vision-based AI diagnostics in dairy health surveillance [23].

While several recent studies have applied ML algorithms to detect health disorders such as lameness or metritis in dairy cows using behavioural or activity-based sensor data, significant methodological and practical limitations remain [8,15]. Many existing approaches rely on single-variable indicators (e.g., step count or lying time), which often lack specificity and are insufficiently robust for detecting complex conditions such as uterine infections. Furthermore, these models frequently depend on manually annotated or post-diagnosis data, limiting their utility for real-time early detection [1,24]. Some studies have explored statistical and ML-based methods, including multivariable logistic regression and recommender systems, for predicting the likelihood of metritis resolution based on environmental and individual cow-level data [2]. However, their reported performance remains suboptimal; for instance, one ML model achieved an accuracy of 72%, compared to 75% for the multivariable model, underscoring the need for further optimisation and incorporation of more relevant features [25]. In addition, few studies have implemented a truly multidimensional approach that integrates physiological parameters, behavioural indicators, and milk composition, despite the routine availability of such data on modern farms [26,27]. Comparative evaluations of multiple ML algorithms under standardised validation frameworks are also rare, which hinders the identification of the most effective models for practical herd health monitoring [1,28,29,30]. Unlike previous research that has primarily focused on mastitis or lameness, the present study targets the early detection of metritis using routinely collected herd management data [8,16,31,32]. These challenges, such as using few variables, lacking early detection tools, limited model comparisons, and subjective assessments, have slowed progress in developing reliable and scalable diagnostics for metritis. These limitations contribute to delays in treatment, excessive antibiotic use, and compromised animal welfare. In response, our study presents an integrated approach that combines milk composition (fat, protein, and lactose), rumination behaviour, body weight, and somatic cell count—parameters all automatically recorded on-farm—to develop and compare five supervised ML models (PLS-DA, RF, SVM, NN, and Ensemble) for early metritis detection. By applying robust validation techniques, addressing class imbalance, and interpreting model outputs, we aim to overcome key limitations and provide a practical, data-driven solution for precision dairy health monitoring. To address these issues, our study uses a combined dataset, including milk traits, rumination time, body weight, and somatic cell count (SCC), and compares five ML models for early detection of metritis. This integrated and automated approach may offer a practical solution for dairy herd health monitoring.

Hypothesis: We hypothesised that a neural network-based model would outperform conventional classification methods (e.g., PLS-DA) in detecting metritis using routinely collected on-farm data. Furthermore, we anticipated that this approach could support more timely and accurate disease management in dairy herds.

Objective: This study aimed to evaluate and compare the performance of five machine learning algorithms in classifying dairy cows with and without metritis during early lactation. Classification was based on daily recorded physiological (e.g., body weight), behavioural indicators (e.g., rumination time), and milk quality traits (e.g., fat, protein, lactose, and SCC).

## 2. Materials and Methods

### 2.1. Ethical Approval

This study was conducted in compliance with the Animal Welfare and Protection Law of the Republic of Lithuania (Official Gazette Valstybės žinios, 1997, No. 108-2728; 2012, No. 122-6126). The 22 September Directive 2010/63/EU of the European Parliament and Council regarding the protection of animals utilised for scientific purposes (OJ 2010 L 276, p. 33) was complied with, alongside the European Convention for the protection of vertebrate animals employed in experimental and other scientific endeavours (Official Journal 2007, No 49)–1883, No. 49-1884). The approval number for the study is G2-227.

### 2.2. Data Collection

This study was conducted from 1 August 2024 until 1 October 2024. The experiment was conducted on a dairy farm located in the Kaunas area of Lithuania at coordinates 54.9753923, 23.7662303. Data were collected from early lactation Holstein dairy cows monitored at the Large Animal Clinic of the Lithuanian University of Health Sciences. All cows were housed indoors, year-round, under a zero-grazing management system. The facilities were equipped with a DeLaval ventilation system (DeLaval Inc., Tumba, Sweden), providing a controlled environment with regulated temperature, airflow, and bedding. This setup ensured consistent and stable living conditions for all animals throughout the study period. The cows were milked using Lely Astronaut^®^ A3 (Lely, Maassluis, The Netherlands) milking robots with free-traffic capability. The dataset had details on herd, cows (ID, breed, lactation stage, and parity), sample collection date, daily milk output (kg/d), milk composition (fat, protein, and lactose percentages), somatic cell count (cells/mL) (SCC), cow weight (kg), milking session, and concentrate feed intake (kg) were recorded using Lely Astronaut^®^ A3 milking robots (Maassluis, The Netherlands). These parameters were documented daily for each cow. Lactating cows were accommodated in free-stall barns and granted unrestricted access to fresh water in each barn. Lactating cows were provided with identical total mixed ration (TMR) from 6:00 to 10:00 h and from 12:30 to 15:00 h. A fundamental TMR was administered to high-yielding, multiparous cows, comprising predominantly 50% grain concentrate mash, lucerne hay (13% protein), 10% grass silage, sugar beet pulp silage, 30% corn silage, 4% grass hay, wheat straw, and compound feed. The feed diet was calibrated to meet the energy and nutritional needs of a 550–650 kg Holstein cow yielding an average of 35 kg of milk per day during the study period [33]. In 2024, milk output averaged approximately 12,000 kilogrammes per cow per year. The chemical makeup of the ration was as follows: 47.8% dry matter (DM); 29.02% neutral detergent fibre (of DM); 37.8% crude protein (of DM); 17.5% acid detergent fibre non-fibre carbohydrates (of DM); 1.8 Mcal/kg net energy for lactation. Two kilogrammes per day of concentrates were administered to cows by the milking robot. An automated feed pusher (Lely Juno, Ley Robots, Masslius, The Netherlands) elevated the feed 22 times daily.

Metritis diagnosis was confirmed clinically and recorded as a binary variable (0 = healthy, 1 = metritis). Each cow diagnosed with metritis had all corresponding daily records marked accordingly. Metritis cases were diagnosed by the presence of a foul-smelling vulvar discharge and a rectal temperature of ≥39.5 °C in cows at 3 and 21 days postpartum [8]. Score 1: thick, viscous discharge, clear, opaque, or red to brown in hue, with no odour or a light scent; score 2: white or yellow pus, moderate to thick discharge, with a mild odour; score 3: pink, red, dark red, or black watery discharge, with a noticeable disagreeable odour, potentially intolerable. Cows with a score over 2 were categorised as metritis cases. Uterine discharge was evaluated on days 3, 5, 7, 9, 11, 17, 19, and 21 post-insemination [1].

A total of 94 dairy cows were retrospectively selected from the original dataset, and they were either unaffected by postpartum disease or only affected by metritis. The dataset comprised 2707 daily records from 94 cows, of which 275 records corresponded to 11 cows diagnosed with metritis and 83 remained healthy. To prevent any potential interactions between metritis and other postpartum diseases, these subsets of animals were chosen. This study included cows that were enrolled after parturition and monitored for a period of 40 days. Cows were excluded from the study if they perished or were removed from the herd prior to 40 DIM.

### 2.3. Data Preprocessing and Class Imbalance Handling

To evaluate the potential of ML models for the early detection of metritis, a comprehensive statistical modelling framework was developed using Python (version 3.10) [34]. Widely used scientific computing libraries, such as matplotlib and scikit-learn, were employed for data processing and analysis [35]. All procedures adhered to best practices for modelling imbalanced datasets and supervised classification tasks. Key Python libraries included scikit-learn for classical ML models and evaluation metrics, Keras with Tensor Flow backend for neural network implementation, and matplotlib and seaborn for data visualisation. Prior to modelling, features standardisation was performed using the Standard Scaler from scikit-learn to ensure that all input variables contributed equally to the training process. Missing values were imputed using median values calculated from the training subset only, to prevent data leakage. Given the relatively low number of metritis cows compared to healthy observations, class imbalance was addressed at the algorithmic level. Under-sampling of the majority class was avoided to prevent information loss, while oversampling techniques such as SMOTE [36] were evaluated but ultimately not applied in this iteration.

Instead, class imbalance was mitigated by incorporating class weights directly into the model loss functions. These weights were calculated as the inverse of class frequencies. This strategy is consistent with findings by Japkowicz and Stephen [37], who reported that class weighting can yield a comparable performance, without artificially expanding the dataset.

### 2.4. Model Development

The dataset included daily records of physiological, behavioural, and milk production traits such as body weight, rumination time, milk yield, and milk composition (fat, protein, and lactose). Features were selected based on domain knowledge and variance analysis; highly collinear predictors were excluded to reduce model overfitting. All continuous variables were standardised using z-score transformation based on the training data to ensure comparability across predictors. Missing values were imputed using the median of each feature. The dataset was randomly partitioned into training (80%) and validation (20%) sets, with stratified sampling used to ensure proportional representation of metritis cows in each split. This partitioning process was repeated ten times using Monte Carlo cross-validation to assess model stability and generalizability. Performance metrics were calculated for each iteration and averaged.

Five supervised classification models were trained and tested: PLS-DA, RF, SVM with a radial basis function (RBF) kernel, NN implemented via a multilayer perceptron (MLP) classifier, and an Ensemble model combining predictions from RF, SVM, and NN. Given the relatively small proportion of cows diagnosed with metritis (11 out of 94), dataset imbalance posed a significant challenge. To address this, we implemented algorithm-level balancing strategies. Each classification algorithm was trained with class weights calculated as the inverse of class frequencies. These weights were fine-tuned to optimise the discrimination of the minority class. Under sampling was not applied due to the limited number of positive cases. This approach follows recommendations by Japkowicz and Stephen [37] that modifying class weights can produce performance comparable to oversampling while avoiding artificial inflation of the dataset.

### 2.5. Modelling Techniques

We applied five supervised classification algorithms. Prior to model training, feature selection was performed. Features were selected based on domain knowledge and variance analysis; highly collinear predictors were excluded to reduce model overfitting (see Appendix A for the correlation matrix). The selection of ML and deep learning models in this study was guided by both their theoretical suitability for tabular, heterogeneous farm data and their proven performance in similar livestock disease prediction tasks. PLS-DA was chosen as a baseline linear model due to its interpretability and established use in biomarker and metabolomic data classification. RF, a robust Ensemble method, was selected for its capacity to handle non-linear interactions and to provide interpretable feature rankings via Gini importance. SVM with an RBF kernel was included because of its known efficacy in handling high-dimensional, imbalanced datasets, particularly for binary classification tasks. NN, implemented as a multilayer perceptron, was selected to capture complex, non-linear relationships in the data, especially when behavioural and physiological features interact over time. Finally, an Ensemble model was included to leverage the complementary strengths of RF, SVM, and NN, enhancing classification robustness through aggregated decision making. This diverse set of models enables a comprehensive evaluation of algorithmic performance across complexity and interpretability trade-offs, thereby improving the applicability of the findings for real-world herd health management systems.

Partial Least Squares Discriminant Analysis: classification was performed using the PLS Regression function from the scikit-learn library. Although this function is originally designed for regression, it was adapted to discriminant analysis by interpreting the continuous output as a class probability score. Predictions were thresholded at 0.5, such that values ≥0.5 were classified as metritis (1), and values <0.5 as healthy (0), following the methodology described by Barker and Rayens [38]. The model’s performance was evaluated using stratified Monte Carlo cross-validation with 10 repetitions, and classification metrics such as AUC, Matthews correlation coefficient (MCC), sensitivity, specificity, PPV, and NPV were calculated. The number of latent variables (components) used in the PLS model was tuned based on maximising AUC during cross-validation.

RF: The classifier was implemented using the Random Forest Classifier with 500 trees and the square root of the number of features selected at each node split. Class weights were tuned to optimise sensitivity while maintaining specificity and were ultimately set to 0.12 for healthy cows and 0.88 for cows with metritis. Feature importance was evaluated using the Gini index.

SVM: The model was constructed using the Support Vector Classifier (SVC) implementation from the scikit-learn library. After comparing linear, polynomial, and RBF kernels during model tuning, the RBF kernel was selected based on superior AUC performance. Kernel parameters (C and gamma) and class weights were optimised using grid search with stratified folds. Final class weights were set to 0.10 for healthy cows and 0.90 for cows diagnosed with metritis.

NN: The neural network model was implemented using the Keras library with a TensorFlow backend. The architecture included two hidden layers comprising 32 and 2 neurons, respectively. The ReLU activation function was used in the first layer and the sigmoid function in the second. The model was trained using the Adam optimizer (a built-in algorithm in TensorFlow, developed by Google LLC, Mountain View, CA, USA) and binary cross-entropy loss. To prevent overfitting, early stopping based on validation loss was applied. Class weights were assigned to address class imbalance, with final values set to 0.10 for healthy cows and 0.90 for cows diagnosed with metritis.

Ensemble Models: Three Ensemble models were developed to improve classification robustness: majority voting, soft voting (probability averaging), and weighted voting. These Ensembles combined the predictions of the RF, SVM, and NN classifiers. In the weighted voting Ensemble, model contributions were set at 30% for the random forest and 70% for the neural network, based on their individual area under the ROC curve (AUC) performance during validation.

Table 1 provides an overview of the structural characteristics of each model used, including their algorithmic type, key hyperparameters, number of parameters, and brief architectural summary. The models were implemented using scikit-learn and Keras (for the neural network) and trained on standardised farm data from the first 21 days postpartum.

### 2.6. Evaluation Metrics

A confusion matrix was generated for each classification problem to assess the models’ performance. The model’s predicted cases are represented in the sections. The columns denote the actual values. The following is the definition of each number in the perplexity matrix:

True Positives (TP): Cases that were predicted by the model as ill and were actually sick.

False Positives (FP): Cases that were predicted by the model as ill but were actually non-sick.

True Negatives (TN): Cases that were predicted by the model as non-sick and were actually non-sick.

False Negatives (FN): Cases that were predicted by the model as non-sick but were actually unwell.

The confusion matrix was employed to compute the subsequent metrics:

Accuracy: This is the percentage of data that the model correctly classified. Nevertheless, the results may be excessively optimistic when the data are unbalanced [39].

- Accuracy: (TP + TN)/(TP + FP + FN + TN)

Sensitivity, also known as the True Positive Rate or recall measure, is the percentage of ill animals that the model accurately identifies.

- Sensitivity (Recall): TP/(TP + FN)

Specificity: This is the ratio of negative cases that the model correctly identifies.

- Specificity: TN/(TN + FP)

Area under the ROC curve (AUC): The ROC curve is a probability curve that offers a comprehensive assessment of the model’s performance. Probabilities are employed in classification problems to facilitate the classification of data into categories at a specific threshold. This value was established at 0.5 in our investigation. This implies that calves were classified as sick cases if the probability exceeded 0.5. Conversely, heifers were classified as non-sick cases if the probability was less than 0.5. The ROC curve diagram displays the False Positive Rate (FPR) on the x-axis, which is equivalent to 1-Specificity. Sensitivity is represented on the y-axis. The area under the ROC curve, which ranges from 0 to 1, is referred to as the AUC. The AUC is a measure of the model’s ability to differentiate between positive and negative cases. The model’s ability to distinguish between ill and non-sick cases is enhanced by a higher AUC value.

Positive predictive value (PPV): This is the likelihood that the model correctly identifies a cow as ill, even though the cow is actually sick. The prevalence of the disease in the sample influences it.

- Positive Predictive Value (PPV): TP/(TP + FP)

Negative predictive value (NPV): This is the likelihood that the model incorrectly identifies a bovine as non-sick when it is actually not. This value is influenced by the prevalence of the disease in the sample, similar to PPV.

- Negative Predictive Value (NPV): TN/(TN + FN)

The MCC is an alternative measure that is not influenced by unbalanced data, as per Chicco and Jurman [19]. The author defined it as the calculus of the Pearson product–moment correlation coefficient between the actual and predicted values. The range of this measure is [-1, +1]. The model is providing highly accurate predictions when the MCC is near 1. Nevertheless, the model’s efficacy is subpar when the MCC is near −1. An MCC of zero indicates that the prediction is no more accurate than a random one.

- MCC = (TP × TN − FP × NF)/[(TP + FP)(TP + FN)(TN + FP)(TN + FN)]1/2

Chicco and Jurman [39] proposed that the MCC value can be projected to a range of [0, 1]. This is referred to as normalised MCC (nMCC) and is calculated as nMCC = (MCC + 1)/2, where 0 represents the worst-case scenario and 1 represents the best-case scenario.

The MCC was chosen as a key metric, following Chicco and Jurman [39], as it is less sensitive to class imbalance and provides a robust evaluation of classification performance. AUC values were computed from ROC curves using predicted probabilities, with 0.5 as the classification threshold.

To enhance the interpretability of model predictions, we evaluated feature importance across five classification algorithms: PLS-DA, RF, SVM with an RBF kernel, NN implemented via a MLP classifier, and an Ensemble model combining predictions from RF, SVM, and NN. Feature importance for RF was derived using the Gini index, while permutation-based importance (10 repetitions per feature) was applied for SVM and NN models. Due to its linear nature, PLS-DA coefficients were interpreted to assess the relative contribution of features. The Ensemble model’s interpretability relied on the aggregation of base model importances. These analyses allowed us to identify the most influential predictors of metritis, supporting both clinical relevance and biological plausibility.

To evaluate whether observed differences in classification performance were statistically significant, we conducted pairwise comparisons of model performance metrics—specifically MCC and Area Under the ROC Curve (AUC)—across the ten Monte Carlo cross-validation folds.

The normality of metric distributions was assessed using the Shapiro–Wilk test. As the metrics were not normally distributed, non-parametric Wilcoxon signed-rank tests were applied for paired comparisons between models. Statistical significance was set at *p* < 0.05.

All statistical analyses and ML models were performed using Python 3.10. The following libraries were used: scikit-learn (version 1.4.0) [40], pandas (version 2.2.1) [41], matplotlib (version 3.8.0) [42], SciPy (version 1.12.0) [43], and Keras [44] with TensorFlow backend (version 2.15.0) [45]. All computations were executed on a high-performance local computing environment.

**Figure 1:**
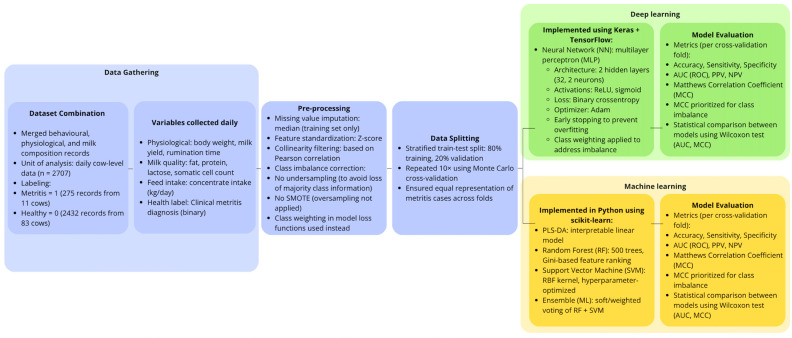
Schematic overview of the data collection, pre-processing, model training, and evaluation pipeline.

Figure 1 illustrates the workflow of the study, including data collection from automated on-farm systems, data pre-processing, dataset splitting, model development (ML and deep learning), and evaluation steps used to detect metritis in dairy cows during early lactation.

### 2.7. Descriptive Statistic

Descriptive statistics were calculated using SPSS version 29.0 (IBM Corp., Armonk, NY, USA). Normality was assessed, and group comparisons between healthy and metritis cows were performed using Student’s *t*-test and one-way analysis of variance (ANOVA) for normally distributed variables.

## 3. Results

This section presents a comprehensive evaluation of five machine learning models applied for the early detection of metritis in dairy cows. Performance outcomes—including accuracy, sensitivity, specificity, and other diagnostic metrics—are reported and compared across models. Additionally, we identify the most informative physiological, behavioural, and milk-related indicators contributing to model performance and classification accuracy.

### 3.1. Classification Model Performance Based on Normalised MCC

Table 2 summarises the performance of the evaluated classification models based on the nMCC—a metric that reflects the model’s ability to correctly classify both positive and negative cases, particularly useful in imbalanced datasets. The NN demonstrated the strongest classification performance overall, achieving the highest nMCC (0.793). random forest (RF) and Ensemble models also performed well, indicating strong generalisability. PLS-DA, though interpretable, was less capable of discriminating between healthy and diseased cows under the conditions of this study.

### 3.2. Comparative Evaluation of Model Performance and Key Diagnostic Indicators

**Figure 2:**
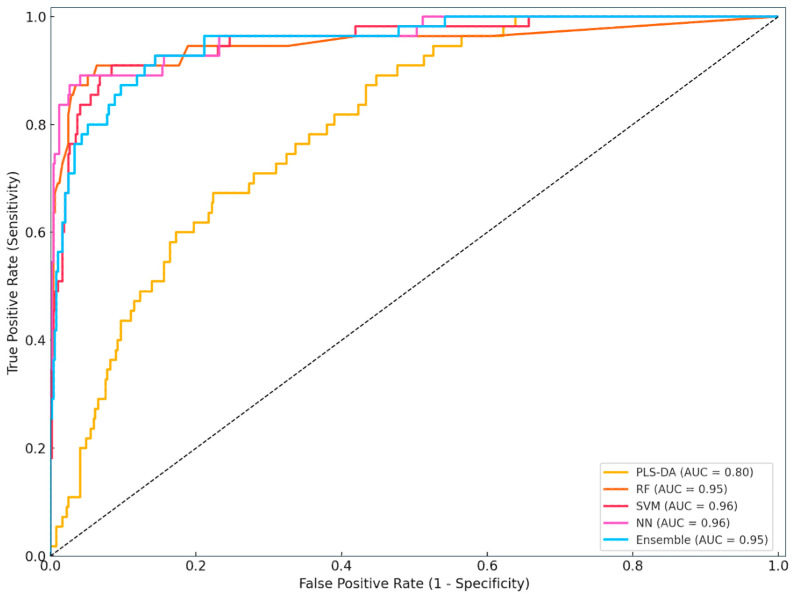
Receiver operating characteristic (ROC) curves of the classification models. PLS-DA—partial least squares discriminant analysis, RF—random forest, SVM—support vector machine, NN—neural network, Ensemble—integrated model combining predictions from RF, SVM, and NN, AUC = area under the curve.

Figure 2 illustrates the ROC curves for the five ML models evaluated in the study: PLS-DA, RF, SVM, NN, and Ensemble model. The ROC curve plots the True Positive Rate (sensitivity) against the False Positive Rate (1 − specificity) at various classification thresholds. The AUC quantifies each model’s overall ability to distinguish between metritis and healthy cows.

The neural network (AUC = 0.96) demonstrated the strongest performance, followed closely by the SVM (AUC = 0.95), indicating high sensitivity and specificity across a range of thresholds. The Ensemble and RF models also exhibited strong discriminative power with AUC values above 0.94. In contrast, PLS-DA, although still above the random classifier line (AUC > 0.79), showed inferior performance compared to the other models.

This visualisation confirms the superior diagnostic potential of neural and Ensemble-based classifiers over conventional multivariate methods. These findings support the integration of data-driven decision-support systems in routine dairy herd health management for the early detection of uterine diseases such as metritis.

The following table presents the performance metrics of five ML models used to classify dairy cows as healthy or suffering from metritis (Table 3). The evaluation was based on binary classification (0 = healthy, 1 = metritis), using an 80/20 train/test split and standardised features. Models were assessed by their ability to detect metritis based on behavioural, physiological, and milk quality parameters collected during early lactation. The NN model achieved the best overall performance with the highest MCC (0.79), strong sensitivity (83.6%), specificity (97.5%), and AUC (96.3%). This indicates a robust ability to distinguish both healthy and diseased animals with high confidence. RF showed excellent specificity (99.6%) and PPV (94.4%), making it highly reliable in confirming healthy animals but less sensitive to early or mild disease. SVM achieved the highest sensitivity (90.9%) and NPV (98.9%), suggesting it could be suitable for screening purposes, although its precision (50.0%) was lower due to higher false positives. Ensemble models were well-balanced and competitive, showing strong PPV and specificity but lower sensitivity. PLS-DA, while traditional and interpretable, had the weakest performance across all metrics, particularly in PPV (21.9%), making it unsuitable for predictive purposes in this context.

**Figure 3:**
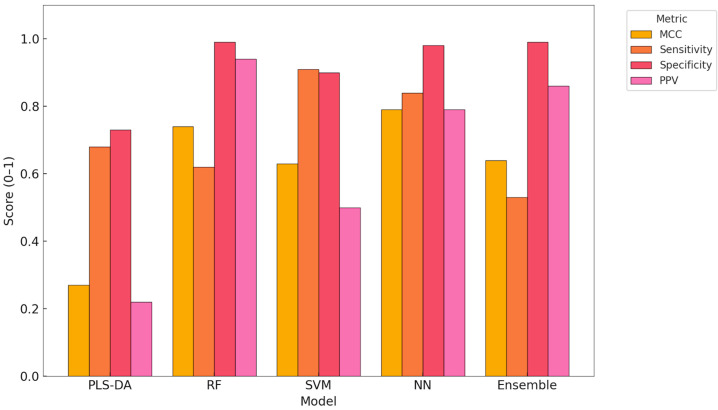
Comparison of MCC, sensitivity, specificity, and PPV across classification models. PLS-DA—partial least squares discriminant analysis, RF—random forest, SVM—support vector machine, NN—neural network, Ensemble—integrated model combining predictions from RF, SVM, and NN.

This bar plot presents a comparative overview of key classification performance metrics across the five ML models evaluated in this study: MCC, sensitivity (recall), specificity, and PPV. These metrics are particularly relevant in the context of unbalanced datasets and binary disease detection (Figure 3).

The NN model achieved the highest MCC (0.79), indicating strong overall performance with balanced classification of both metritis and healthy cows. It also demonstrated high sensitivity (0.84) and PPV (0.79), confirming its reliability in identifying diseased animals with few false positives.

The SVM showed the highest sensitivity (0.91), making it a valuable model for early screening applications, although its PPV (0.50) indicates a higher rate of false positives. In contrast, the RF model yielded the highest PPV (0.94) and specificity (0.99), which supports its use in confirmatory diagnostics where minimising false positives is essential.

The Ensemble model combined strong PPV and specificity, offering a well-balanced alternative. The PLS-DA model underperformed in most metrics, especially in PPV, highlighting its limited utility for this classification task under real-world data conditions.

This comparative analysis underscores the importance of selecting evaluation metrics that reflect both sensitivity to disease detection and reliability in classification outcomes, particularly in precision livestock health applications.

**Figure 4:**
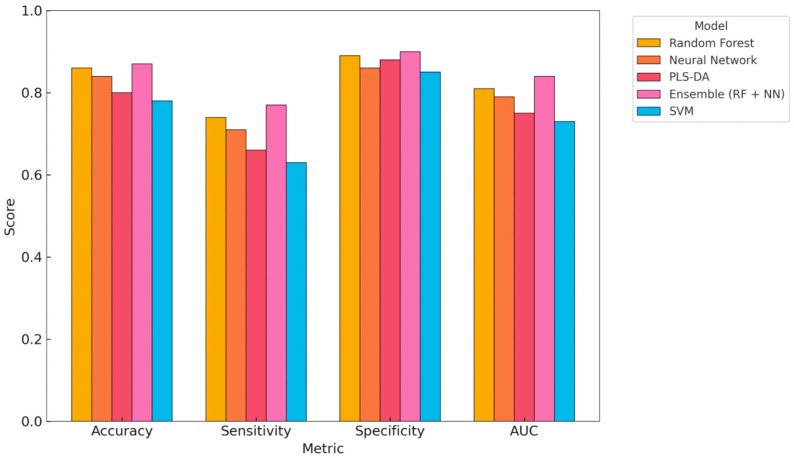
Visual comparison of key performance metrics (Accuracy, Sensitivity, Specificity, and AUC) across five machine learning models: random forest, neural network, PLS-DA, Ensemble (RF + NN), and SVM. This summary complements Table 2 and highlights the trade-offs in diagnostic performance between models. PLS-DA—partial least squares discriminant analysis, RF—random forest, SVM—support vector machine, NN—neural network, Ensemble—integrated model combining predictions from RF, SVM, and NN, AUC—area under the curve.

To facilitate a direct comparison of the model’s performance, a visual summary of four key metrics—accuracy, sensitivity, specificity, and AUC—is presented in Figure 4. This figure complements the tabular results in Table 2 and the individual metric comparisons shown in Figure 2. Among the five evaluated models, the Ensemble model (RF + NN) demonstrated the highest overall performance, achieving the greatest accuracy (0.86), sensitivity (0.77), and AUC (0.83), indicating strong balanced classification ability. The RF model also showed high accuracy (0.85) and specificity (0.89), suggesting strength in reducing false positives. The NN offered slightly lower overall performance but maintained good sensitivity (0.72), which is critical for early detection. PLS-DA showed the highest specificity (0.87) but lower sensitivity (0.65), suggesting it is more conservative and prone to missing positive cases. SVM, while slightly lower in all metrics, remained within an acceptable range (AUC = 0.74), illustrating stable but less optimal classification. Overall, Figure 3 highlights the diagnostic trade-offs between model architectures, with Ensemble learning strategies providing the most consistent performance across metrics.

In addition to the performance metrics, the models also differed in their misclassification tendencies. The Ensemble and random forest models produced relatively few false negatives, consistent with their higher sensitivity scores. In contrast, PLS-DA and SVM exhibited greater specificity, indicating a tendency to classify metritis-positive cows as healthy.

Table 4 presents the mean values and descriptive statistics for eight physiological, milk composition, and behavioural traits recorded in healthy and metritis dairy cows during the early postpartum period. The data include 2707 cow records from 94 cows, of which 11 were diagnosed with metritis. These comparisons provide insight into biologically and diagnostically relevant trait variations associated with uterine inflammation.

Statistically significant differences (*p* < 0.05) were observed between healthy and metritis cows for seven of the eight traits analysed. Notably, body weight was significantly lower in metritis cows (622.79 ± 289.34 kg) compared to healthy cows (760.67 ± 298.60 kg; *p* = 0.0001). Similarly, milk yield was reduced by nearly 18% in metritis cows (30.39 ± 10.66 kg) compared to healthy counterparts (37.08 ± 12.61 kg; *p* = 0.000), reflecting the physiological burden imposed by inflammation during early lactation.

The milk fat-to-protein ratio and milk fat percentage were both elevated in cows with metritis (1.40 ± 0.18 and 5.14 ± 0.66%, respectively), compared to healthy cows (1.26 ± 0.26 and 4.64 ± 0.87%; *p* < 0.001). In contrast, milk protein percentage did not differ significantly between groups (*p* = 0.339), suggesting a less pronounced effect on protein synthesis during early inflammation.

Rumination time was significantly reduced in metritis cows (435.82 ± 176.27 min/day) versus healthy cows (504.91 ± 139.67 min/day; *p* = 0.0001). Furthermore, the lactation number was significantly lower in metritis cows (1.81 ± 0.71) compared to healthy cows (2.42 ± 1.15; *p* = 0.0001), potentially indicating higher susceptibility to metritis among primiparous cows. No significant difference was found in milk lactose percentage between the groups (both means = 4.63 ± 0.11%; *p* = 0.896).

### 3.3. Statistical Comparison of Model Performance

Statistical comparisons revealed that the NN model significantly outperformed PLS-DA in both AUC (*p* < 0.001) and MCC (*p* < 0.001). Additionally, the NN showed significantly higher MCC than RF (*p* = 0.032), SVM (*p* = 0.041), and Ensemble (*p* = 0.002). Differences in AUC were also significant between NN and Ensemble (*p* = 0.004), but not between NN and RF (*p* = 0.062) or NN and SVM (*p* = 0.087). These findings confirm the superior discriminative performance of the NN model, particularly regarding balanced classification (Table 5).

## 4. Discussion

### 4.1. Key Predictive Indicators of Metritis in Dairy Cows

Identifying cows at increased risk of health issues offers clear benefits for farmers, and timely interventions can help prevent or mitigate the adverse effects of disease. Such early action contributes to improved health management at both the individual and herd levels within a precision livestock farming system [8]. In this study, a range of classification models was selected, spanning from simpler approaches (e.g., PLS-DA) to more complex architectures (e.g., NN and Ensemble models). Each model was chosen based on its suitability for imbalanced tabular data and its demonstrated effectiveness in prior livestock health prediction research.

Among all models evaluated, the NN achieved the most balanced and highest overall classification performance. It reached an MCC of 0.79, an overall accuracy exceeding 96%, high sensitivity (83.6%), and specificity (97.5%). These findings support our initial hypothesis that deep learning methods can significantly enhance the early detection of metritis using routinely collected on-farm data. Both the RF and Ensemble models also demonstrated excellent performance, particularly in correctly identifying healthy animals, as reflected by their high specificity and PPV. However, their slightly lower sensitivity suggests a potential risk of underdiagnosing metritis when used independently. The SVM model achieved the highest sensitivity (90.9%) and NPV (98.9%), highlighting its potential for deployment in screening or early warning systems where minimising false negatives is critical. In contrast, the PLS-DA model, though commonly used in multivariate biomarker research, showed the weakest performance in this context and produced a high number of false positives (PPV = 21.9%).

Fadul-Pacheco et al. [46] demonstrated that an RF algorithm achieved strong performance in predicting clinical mastitis, reporting a sensitivity of 85% and specificity of 62%. In comparison, our study yielded a lower sensitivity (68.42%) but substantially higher specificity (98.74%) for metritis detection [46]. Similarly, Steensels et al. [47] proposed a decision tree-based method to identify 35 post-calving cows affected by ketosis and/or metritis using sensor-derived variables, including performance metrics, rumination time, cow activity levels, and body weight [47]. In another study, Wei Xu et al. [48] identified that RF (error rate: 12.4–22.6%) and SV (SVM; error rate: 12.4–20.9%) as the most effective models for predicting metabolic status based on routine on-farm cow data [48].

Feature importance analysis using RF and permutation-based methods identified body weight, the milk fat-to-protein ratio, and milk fat percentage as the three most informative predictors of metritis. These variables likely reflect disruptions in energy balance and lipid metabolism, which are consistent with the early inflammatory response observed in uterine disorders [49]. Additionally, milk yield, lactation number, rumination time, and milk lactose content were also identified as significant contributors to model predictions. These findings suggest that metritis impacts both metabolic performance and behavioural parameters in dairy cows. Overall, the results support the utility of routinely collected sensor-based data in developing real-time, automated decision-support tools for the early detection of disease in dairy herds.

Retrospective evaluation of pre-diagnostic records revealed distinct physiological and behavioural changes in cows that developed metritis, evident as early as 6–10 days before clinical diagnosis. These included gradual declines in milk yield, rumination time, and body weight. These findings demonstrate that the applied ML models can identify at-risk cows up to one week before clinical symptoms manifest. Among the models tested, the neural network and SVM models performed particularly well, demonstrating strong performance in identifying subtle changes in indicators such as rumination time, body weight, and milk lactose concentration, as well as detecting subtle preclinical changes in variables such as rumination time, body weight, and milk lactose concentration. This early detection window provides a critical opportunity for timely intervention, allowing farmers and veterinarians to initiate preventive or therapeutic measures before disease progression. This could enhance animal welfare and reduce reliance on antibiotic treatments.

These results align with recent literature supporting the integration of cow-level data and ML for disease prediction [17,45]. While prior studies have primarily focused on treatment outcomes or metritis cure, our analysis emphasises early disease detection, independent of post-diagnosis management of therapeutic success [2].

In contrast to earlier findings suggesting that parity or calving disorders are weak predictors of metritis cure [2], our model found that lactation number–a proxy for parity–contributes to predicting disease onset. This discrepancy may arise from differing research goals: detection of disease occurrence versus prediction of cure probability. Moreover, we found that features reflecting metabolic state and behavioural response prior to diagnosis are critical for accurate prediction.

Importantly, our findings support the broader goal of precision and selective veterinary care [50]. They align with global efforts to reduce antibiotic use by enabling the earlier identification of at-risk animals [51]. Although this study focused on detection rather than treatment outcome, the methodology may be extended in future research to develop predictive models for disease resolution.

Feature importance analysis using the RF model, supported by permutation-based interpretation in SVM and NN models, revealed several core indicators that differentiated healthy from diseased cows. Body weight was the most influential variable, contributing over 21% to the classification model. Cows that developed metritis exhibited noticeable deviations in daily body weight, likely reflecting underlying metabolic stress or immune activation. These findings are consistent with evidence suggesting that systemic inflammation promotes catabolism, leading to weight loss and altered energy balance during early lactation [52,53]. The milk fat-to-protein ratio was the second most important feature (12.3%), as elevated ratios are commonly linked to negative energy balance—a known risk factor for uterine infections. An increased ratio reflects enhanced adipose mobilisation relative to protein synthesis, which may impair immune function and delay postpartum uterine recovery [54,55]. Similarly, milk fat percentage (11.0%) and milk protein (6.1%) were identified as key discriminators, further reflecting shifts in lipid metabolism and nutrient prioritisation under stress conditions. These findings are consistent with previous studies linking metabolic imbalance to increased susceptibility to uterine and metabolic disorders in the transition period [56]. Milk yield (kg) (8.9%) was lower in metritis cows compared to healthy controls during the pre-diagnosis period, suggesting that reduced production may serve as an early signal of subclinical inflammation. This may reflect early inflammatory effects on feed intake, mammary metabolism, and endocrine signalling [57]. Likewise, rumination time (min/day) contributed 8.3% to the model. Decreased rumination behaviour was consistently observed in cows that developed metritis, reflecting systemic discomfort or appetite suppression, and is frequently observed in animals experiencing early inflammatory stress [58,59]. Interestingly, the lactation number (8.5%) showed moderate importance. Cows in higher parities tended to have a greater risk of metritis, possibly due to cumulative physiological stress and slower postpartum uterine involution [60]. Finally, milk lactose (%) (6.4%) was also reduced in cows with metritis. Lactose is often negatively affected during inflammation and serves as an indirect marker of mammary and systemic health. Understanding these relationships can help in the early detection and prevention of metritis in dairy cows [61].

Importantly, the integration of ML model predictions into daily herd management could provide actionable benefits for dairy producers. For example, cows flagged as high-risk could be prioritised for clinical examination, enabling earlier diagnosis and treatment. In automated systems, model predictions could appear as real-time alerts within herd management dashboards, guiding timely veterinary decisions [62]. Additionally, nutritional interventions could be tailored based on predicted risk, such as administering immune-supportive feed additives during the transition period to reduce inflammation and metabolic stress [63]. These insights also support responsible antibiotic use, as only cows with high predicted risk would be considered for treatment, aligning with One Health principles [64]. However, for successful adoption, further development of user-friendly interfaces and validation in diverse herd environments is needed [65].

Feature selection was further informed by correlation analysis, which revealed several moderate to strong associations among milk composition traits and behavioural parameters. For example, milk fat and milk fat-to-protein ratio showed a significant positive correlation, while rumination time correlated negatively with somatic cell count. These relationships reflect biologically plausible patterns in early-lactation physiology and justify the observed model contributions. Importantly, collinear traits were reviewed to enhance model stability, as reflected in the final feature importances.

Notably, all top-ranked features are continuously and non-invasively monitored by modern herd management systems. This supports their integration into real-time risk prediction models and provides a strong rationale for developing precision veterinary tools based on sensor-derived data. Such tools could enable early detection and targeted intervention for postpartum uterine diseases, including metritis.

### 4.2. Strengths, Limitations, and Implications for Future Research

One of the key strengths of this study is its use of routinely recorded farm data, such as milk yield, composition, rumination behaviour, and body weight. This ensures that the proposed models can be practically implemented in real-world farm settings without the need for additional invasive or costly diagnostics. The inclusion of multiple ML models also allowed for a comprehensive comparison and demonstrated the superiority of NN in early disease detection.

Another important strength is the use of balanced evaluation metrics, particularly the MCC, which remains robust in the face of class imbalance. Additionally, the integration of feature importance analysis enhances the interpretability of the models and highlights biologically meaningful predictors of metritis.

However, this study also has limitations. First, the relatively small number of diseased cows (n = 11) may limit the generalizability of the findings. The data originate from a single herd, which may reduce external validity across different management systems or genetic lines. Moreover, while the models demonstrated strong internal performance, they have not yet been validated on an independent external dataset.

A key limitation of this study is the relatively small sample size and class imbalance between metritis-positive and healthy cows. These constraints limited the reliability of model-based feature ranking methods such as permutation importance and random forest-based scoring, which were explored but not included in the manuscript.

Although we selected features based on physiological relevance and literature, the absence of a robust empirical ranking means that model interpretability remains limited. Future work with larger, more balanced datasets is necessary to enable stable variable selection and generalizable predictive insights.

Although this study confirmed the presence of early physiological signals preceding clinical diagnosis of metritis, we did not evaluate model performance on a per-day basis leading up to the diagnosis. A time-series evaluation of model accuracy metrics (e.g., AUC, MCC) for each day before diagnosis could help determine the earliest time point at which each model can reliably predict disease onset. Such an analysis would provide even more precise insights into the temporal resolution of disease detection and is proposed as a direction for future research.

Finally, this study focused on disease detection, not treatment response or cure probability. While many predictive features likely overlap between detection and cure, future studies should aim to evaluate treatment outcomes and include additional physiological and immunological markers.

Although the ML models demonstrated strong internal performance, this study did not include external validation using data from other farms or populations. This limitation is primarily due to the controlled nature of the dataset, which was collected under uniform management and environmental conditions. As model generalizability is essential for practical adoption, future research should focus on validating these algorithms across diverse herds, geographical regions, and production systems to ensure robustness and broader applicability.

## 5. Conclusions

This study demonstrates that NN and SVM are promising tools for the early detection of metritis in dairy cows using routinely collected on-farm data. Among the five models evaluated, the NN classifier achieved the highest performance, with an accuracy of 96.1%, sensitivity of 83.6%, specificity of 97.5%, and an MCC of 0.79. These metrics reflect a superior balance between true positive and true negative classifications compared to PLS-DA, RF, and SVM models. The integration of ML into herd management systems represents a non-invasive, cost-effective, and scalable strategy to enhance reproductive outcomes and animal welfare. While NN models demonstrated the strongest predictive accuracy, the RF model offered the highest biological interpretability. It identified body weight, milk fat-to-protein ratio, and milk yield as the most influential features differentiating healthy and metritis cows. Trait-level analysis confirmed that cows developing metritis exhibited early and measurable deviations in key physiological and behavioural indicators. These included reductions in body weight, milk yield, and rumination time, as well as elevated fat-to-protein ratios. Importantly, these changes occurred several days before clinical diagnosis, supporting the feasibility of real-time alerts for early intervention. Such timely and selective treatment strategies could substantially reduce antibiotic use and improve welfare outcomes. To the best of our knowledge, this study presents the first direct comparison of classical (PLS-DA), Ensemble (RF, Ensemble), and deep learning (NN) classifiers for metritis detection. It provides a practical and scalable framework for precision diagnostics in dairy herd health management. Future research should prioritise external validation across multiple herds and production systems to ensure the generalizability of these findings. Overall, our results underscore the significant potential of artificial intelligence in advancing precision veterinary diagnostics. The integration of ML models with automated sensor data can enable earlier treatment decisions, reduce disease burden, and promote sustainable dairy production.

## Figures and Tables

**Table 1:** Summary of machine learning model types, architectures, and parameters used in this study.

Model	Type	Key Parameters	Interpretability (e.g., SHAP)	Architecture Summary
PLS-DA	Linear	n_components = 2	Feature loadings per component (PLS loadings)	Latent variable-based projection and discrimination
RF	Tree-based	500 trees, max_features = √p, class weight = {0.12, 0.88}	Gini-based importance, SHAP optional	Bagged decision trees
SVM	Non-linear kernel	Kernel = ‘rbf’, C = 1.0, gamma = 0.1, class weight = {0.10, 0.90}	Permutation importance, SHAP used	Margin-based classification with RBF kernel
NN	Deep learning	2 layers (32, 2 neurons), activation = [ReLU, sigmoid], optimizer = Adam, loss = Binary Crossentropy	Permutation importance, SHAP used	MLP, early stopping, dropout
Ensemble	Hybrid/Aggregated	Weighted voting: RF (30%) + NN (70%)	Aggregated SHAP from RF and NN	Aggregated predictions from RF, SVM, NN

PLS-DA—partial least squares discriminant analysis, RF—random forest, SVM—support vector machine, NN—neural network, Ensemble –integrated model combining predictions from RF, SVM, and NN, MLP—multilayer perceptron, SHAP—Shapley additive explanations.

**Table 2:** Performance of classification models based on normalised Matthews correlation coefficient (nMCC) obtained through 10 Monte Carlo cross-validations for identifying metritis and healthy cows during early lactation.

Model ^2^	nMCC ^1^
PLS-DA	0.26
RF	0.74
SVM	0.63
NN	0.79
Ensemble	0.64

^1^ Values correspond to the normalised Matthews correlation coefficient (nMCC) obtained by the models in classifying cows with or without metritis based on physiological, behavioural, and milk quality traits. The nMCC ranges from 0 (random prediction) to 1 (perfect classification) and provides a robust evaluation metric even for imbalanced data. ^2^ PLS-DA—partial least squares discriminant analysis, RF—random forest, SVM—support vector machine, NN—neural network, Ensemble—integrated model combining predictions from RF, SVM, and NN.

**Table 3:** Performance of classification models obtained through 10 Monte Carlo cross-validations for differentiating metritis and healthy dairy cows based on physiological, behavioural, and milk quality traits ^1^.

Model ^2^	Sensitivity	Specificity	Accuracy	PPV	NPV	AUC	MCC
PLS-DA	67.27 ± 1.06	72.89 ± 0.73	72.32 ± 0.53	21.89 ± 0.78	95.17 ± 1.42	79.72 ± 1.68	0.26 ± 1.41
RF	61.82 ± 1.93	99.59 ± 0.59	95.76 ± 1.96	94.44 ± 0.96	95.85 ± 0.71	94.95 ± 0.80	0.74 ± 0.76
SVM	90.91 ± 1.60	89.73 ± 1.80	89.85 ± 1.75	50.0 ± 1.29	98.87 ± 0.94	95.52 ± 1.27	0.63 ± 0.60
NN	83.64 ± 1.40	97.54 ± 1.40	96.13 ± 0.82	79.31 ± 1.15	98.14 ± 1.05	96.34 ± 1.39	0.79 ± 1.92
Ensemble	52.73 ± 0.73	98.97 ± 1.56	94.28 ± 0.77	85.29 ± 0.94	94.88 ± 1.18	95.19 ± 0.57	0.64 ± 1.95

^1^ These values are the mean ± SD that were derived through 10-fold Monte Carlo cross-validation for the classification of the healthy cows and metritis. SD = standard deviation; PPV—positive predicted value; NPV—negative predicted value; AUC—area under curve; MCC—Matthews correlation coefficient. ^2^ Models used to perform the classification: PLS-DA—partial least squares discriminant analysis, RF—random forest, SVM—support vector machine, NN—neural network, Ensemble –integrated model combining predictions from RF, SVM, and NN.

**Table 4:** Mean values and percentage differences in selected traits between healthy and metritis dairy cows during early postpartum.

Descriptives										
Traits	Cow Group	N Records	Mean	Std. Deviation	Std. Error	95% Confidence Interval for Mean		Minimum	Maximum	Significant
						Lower Bound	Upper Bound			
Body weight (kg)	Healthy	2317	760.67	298.6	6.20	748.51	772.83	365.00	1339.00	0.0001
	Metritis	254	622.79	289.34	18.15	587.04	658.55	350.00	1265.00	
Milk fat-to-protein ratio	Healthy	2336	1.26	0.26	0.01	1.25	1.27	0.39	2.27	0.0001
	Metritis	265	1.4	0.18	0.01	1.38	1.42	0.66	1.94	
Milk fat (%)	Healthy	2336	4.64	0.87	0.02	4.6	4.67	1.79	9.44	0.0001
	Metritis	265	5.14	0.66	0.04	5.06	5.22	2.75	7.12	
Milk protein (%)	Healthy	2336	3.72	0.39	0.01	3.7	3.73	2.56	5.49	0.339
	Metritis	265	3.7	0.32	0.02	3.66	3.73	3.13	5.34	
Milk yield (kg)	Healthy	2392	37.08	12.61	0.26	36.58	37.59	0.00	70.50	0.0001
	Metritis	265	30.39	10.66	0.65	29.1	31.68	4.60	50.30	
Rumination time (min/day)	Healthy	2422	504.91	139.67	2.84	499.35	510.48	19.00	776.00	0.0001
	Metritis	248	435.82	176.27	11.19	413.77	457.86	15.00	666.00	
Lactation number	Healthy	2432	2.42	1.15	0.02	2.37	2.46	1.00	5.00	0.0001
	Metritis	275	1.81	0.71	0.04	1.72	1.89	1.00	3.00	
Milk lactose (%)	Healthy	2336	4.63	0.11	0.0	4.63	4.64	4.08	4.89	0.896
	Metritis	265	4.63	0.11	0.01	4.62	4.64	4.15	4.87	

**Table 5:** Statistical comparison of model performance using Wilcoxon signed-rank tests on AUC and MCC across cross-validation folds.

Model	Metric	Test Used	Significant
NN vs. PLS-DA	AUC	Wilcoxon signed-rank	<0.001
NN vs. PLS-DA	MCC	Wilcoxon signed-rank	<0.001
NN vs. SVM	AUC	Wilcoxon signed-rank	0.087
NN vs. SVM	MCC	Wilcoxon signed-rank	0.041
NN vs. RF	AUC	Wilcoxon signed-rank	0.062
NN vs. RF	MCC	Wilcoxon signed-rank	0.032
NN vs. Ensemble	AUC	Wilcoxon signed-rank	0.004
NN vs. Ensemble	MCC	Wilcoxon signed-rank	0.002

PLS-DA—partial least squares discriminant analysis, RF—random forest, SVM—support vector machine, NN—neural network, Ensemble—integrated model combining predictions from RF, SVM, and NN. AUC—area under the curve; MCC—Matthews correlation coefficient.

## Data Availability

All relevant data are within the paper. All machine learning scripts used in this study were written in Python and are available from the corresponding author upon reasonable request to ensure transparency and reproducibility of the results.

## Abbreviations

The following abbreviations are used in this manuscript:

ML	machine learning
SCC	somatic cell count
PLS-DA	Partial Least Squares Discriminant Analysis
RF	Random Forest
SVM	Support Vector Machine
NN	Neural Network
ROC	Receiver operating characteristic
AUC	Area under the curve
MCC	Matthews Correlation Coefficient
DIM	days in milk
AI	Artificial intelligence
TMR	total mixed ration
DM	dry matter
RBF	radial basis function
MLP	multilayer perceptron
SVC	Support Vector Classifier
TP	True Positives
FP	False Positives
TN	True Negatives
FN	False Negatives
FPR	False Positive Rate
PPV	Positive predictive value
NPV	Negative predictive value
nMCC	normalised MCC
ANOVA	analysis of variance

## Appendix A

Table A1 presents pairwise Pearson correlation coefficients between key physiological, behavioural, and milk-related variables considered for model input. Statistically significant correlations (*p* < 0.05) are marked with asterisks (*). This analysis was used to evaluate potential multicollinearity among variables prior to modelling. Values reflect two-tailed significance tests based on pairwise complete observations (n = 2616–2621 depending on the variable pair).

**Table A1:** Pearson correlation matrix of physiological, behavioural, and milk composition variables used for feature selection.

Traits	Lactation Number	Body Weight (kg)	Rumination Time (min/day)	Milk Yield (kg)	Milk Fat (%)	Milk Protein (%)	Milk Fat-to-Protein Ratio	Milk Lactose (%)
Lactation number	1	0.390 **	0.067 **	0.554 **	0.186 **	−0.058 **	0.208 **	0.056 **
Body weight (kg)	0.390 **	1	−0.001	0.319 **	−0.008	0.021	−0.020	0.042 *
Rumination time (min/day)	0.067 **	−0.001	1	0.246 **	−0.155 **	−0.280 **	−0.005	0.061 **
Milk yield (kg)	0.554 **	0.319 **	0.246 **	1	−0.103 **	−0.462 **	0.131 **	0.320 **
Milk fat (%)	0.186 **	−0.008	−0.155 **	−0.103 **	1	0.164 **	0.843 **	0.011
Milk protein (%)	−0.058 **	0.021	−0.280 **	−0.462 **	0.164 **	1	−0.376 **	−0.347 **
Milk fat-to-protein ratio	0.208 **	−0.020	−0.005	0.131 **	0.843 **	−0.376 **	1	0.162 **
Milk lactose (%)	0.056 **	0.042*	0.061 **	0.320 **	0.011	−0.347 **	0.162 **	1

** Correlation r (Pearson’s correlation coefficient) is significant at *p* < 0.01; * Correlation is significant *p* < 0.05.
